# Characterization of FKGK18 as Inhibitor of Group VIA Ca^2+^-Independent Phospholipase A_2_ (iPLA_2_β): Candidate Drug for Preventing Beta-Cell Apoptosis and Diabetes

**DOI:** 10.1371/journal.pone.0071748

**Published:** 2013-08-20

**Authors:** Tomader Ali, George Kokotos, Victoria Magrioti, Robert N. Bone, James A. Mobley, William Hancock, Sasanka Ramanadham

**Affiliations:** 1 Department of Cell, Developmental, and Integrative Biology, University of Alabama at Birmingham, Birmingham, Alabama, United States of America; 2 Laboratory of Organic Chemistry, Department of Chemistry, University of Athens, Athens, Greece; 3 Department of Pathology, University of Alabama at Birmingham, Birmingham, Alabama, United States of America; 4 Department of Surgery, University of Alabama at Birmingham, Birmingham, Alabama, United States of America; Broad Institute of Harvard and MIT, United States of America

## Abstract

Ongoing studies suggest an important role for iPLA_2_β in a multitude of biological processes and it has been implicated in neurodegenerative, skeletal and vascular smooth muscle disorders, bone formation, and cardiac arrhythmias. Thus, identifying an iPLA_2_βinhibitor that can be reliably and safely used *in vivo* is warranted. Currently, the mechanism-based inhibitor bromoenol lactone (BEL) is the most widely used to discern the role of iPLA_2_β in biological processes. While BEL is recognized as a more potent inhibitor of iPLA_2_ than of cPLA_2_ or sPLA_2_, leading to its designation as a “specific” inhibitor of iPLA_2_, it has been shown to also inhibit non-PLA_2_ enzymes. A potential complication of its use is that while the *S* and *R* enantiomers of BEL exhibit preference for cytosol-associated iPLA_2_β and membrane-associated iPLA_2_γ, respectively, the selectivity is only 10-fold for both. In addition, BEL is unstable in solution, promotes irreversible inhibition, and may be cytotoxic, making BEL not amenable for *in vivo* use. Recently, a fluoroketone (FK)-based compound (FKGK18) was described as a potent inhibitor of iPLA_2_β. Here we characterized its inhibitory profile in beta-cells and find that FKGK18: (a) inhibits iPLA_2_β with a greater potency (100-fold) than iPLA_2_γ, (b) inhibition of iPLA_2_β is reversible, (c) is an ineffective inhibitor of α-chymotrypsin, and (d) inhibits previously described outcomes of iPLA_2_β activation including (i) glucose-stimulated insulin secretion, (ii) arachidonic acid hydrolysis; as reflected by PGE2 release from human islets, (iii) ER stress-induced neutral sphingomyelinase 2 expression, and (iv) ER stress-induced beta-cell apoptosis. These findings suggest that FKGK18 is similar to BEL in its ability to inhibit iPLA_2_β. Because, in contrast to BEL, it is reversible and not a non-specific inhibitor of proteases, it is suggested that FKGK18 is more ideal for *ex vivo* and *in vivo* assessments of iPLA_2_β role in biological functions.

## Introduction

Phospholipases A_2_ (PLA_2_s) catalyze hydrolysis of the *sn*-2 substituent from membrane phospholipids [1]. To date, 16 distinct groups of PLA_2_s are recognized [2], [3] and they include secretory (sPLA_2_s), cytosolic (cPLA_2_s), and Ca^2+^-independent (iPLA_2_s) enzymes. Of these, the Group VI iPLA_2_s are the most recently described and the least well characterized. The iPLA_2_ was first purified from macrophages in 1994 [4] and subsequently cloned from multiple sources between 1997 and 1999 [5]–[7]. This enzyme localized to the cytosol under basal conditions is designated iPLA_2_β. Subsequently, a membrane-associated enzyme was identified and designated as iPLA_2_γ. More comprehensive reviews of the iPLA_2_ enzymes can be found elsewhere [8]–[12].

Since its original description in heart and pancreas, the iPLA_2_β has been proposed to participate in membrane phospholipid remodeling, signal transduction, cell proliferation, inflammation, and apoptosis [8]–[12]. Its dysregulation has been associated with several neurodegenerative, skeletal, and vascular smooth muscle disorders, bone formation, and cardiac arrhythmias [11]. If we are to gain a greater understanding of the mechanism(s) by which iPLA_2_β contributes to these abnormalities, reagents that more specifically impact iPLA_2_β *in vitro* and *in vivo* are needed. Of the currently used inhibitors, iPLA_2_β is targeted by arachidonyl trifluoromethyl ketone (AACOCF_3_), methyl arachidonyl fluorophosphonate (MAFP), and palmitoyl trifluoromethyl ketone (PACOCF_3_); inhibitors that are sometimes used for “selective” inhibition of cPLA_2_ [13]–[15].

While siRNAs directed at iPLA_2_βand now available iPLA_2_β-KO and Tg mice [16]–[18] have provided insight into biological processes impacted by iPLA_2_β, the majority of studies to assess the role of the iPLA_2_β isoform, have utilized the only available specific inhibitor of iPLA_2_ [11]. This inhibitor, (E)-6-(bromo- methylene) tetrahydro-3-(1-naphthalenyl)-2H-pyran-2-one, was synthesized in 1991 and was designated as a haloenol lactone suicide substrate (HELSS) [19], but is now referred to as bromoenol lactone (BEL). The BEL is an irreversible suicide inhibitor that selectively targets iPLA_2_ enzymes and has little or no effect on cPLA_2_ or sPLA_2_ activity [19]–[21].

Over the years, BEL has been used to discern the involvement of iPLA_2_ in biological processes and, to date, is still considered the only available specific irreversible inhibitor of iPLA_2_. Recently, the *S*- and *R*-enantiomers of BEL have been demonstrated to exhibit specific inhibition of iPLA_2_β and iPLA_2γ_, respectively [22]. However, several examples of inhibition of non-PLA_2_ enzymes by BEL have been described [21], [23]–[26] and the mechanism of inhibition does not appear to involve the active site of iPLA_2_ [27], [28]. Although BEL treatment results in covalent modification of iPLA_2_β [14], [19], the modified residues are cysteines and not the active site serine, likely due to a diffusible bromoketomethyl acid that is generated when iPLA_2_ acts on the inhibitor [28].

**Figure 1:**
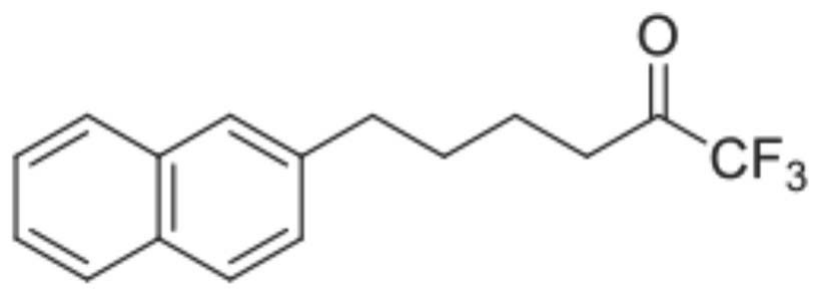
Structure of FKGK18. Chemical structure of 1,1,1-trifluoro-6-(naphthalen-2-yl)hexan-2-one (FKGK18).

However, several features of BEL decrease its feasibility for *in vivo* use: (a) irreversible inhibition of iPLA_2_, (b) inactivation of other serine proteases, and (c) high toxicity due to its interaction with cysteines. For these reasons, recent efforts were directed towards synthesizing alternative compounds that can specifically inhibit iPLA_2_. Assays for PLA_2_ activity in the presence of these compounds have led to the identification of fluoroketone (FK)-based compounds as potential inhibitors of the iPLA_2_ enzyme group [29]. Because FK inhibitors target serine active sites they could potentially also inhibit cPLA_2_s. However, modification of the FK group along with addition of a hydrophobic terminus connected by a medium-length carbon chain to mimic the fatty acid chain conferred selectivity of the FK compounds for iPLA_2_ versus sPLA_2_ or cPLA_2_ [29]. Among the ones tested, FKGK18 (**Fig. 1**) was found to be the most potent inhibitor of GVIA iPLA_2_ and was 195 and >455 times more potent for GVIA iPLA_2_ than for GIVA cPLA_2_ and GV sPLA_2_, respectively.

While the above study demonstrated the potential of FKGK18 in inhibiting iPLA_2_β, the biochemical assays were performed using human Group VIA enzyme purified from Sf9 cells [30]. Thus, it is not known whether FKGK18 is able to inhibit iPLA_2_β in biological systems. Recently, earlier generation of FK compounds (FKGK11 and FKGK2) were found to be effective in ameliorating experimental autoimmune encephalomyelitis, however, there was no demonstration of iPLA_2_β inhibition by these compounds [31]. Our work reveals that iPLA_2_β is predominantly expressed in pancreatic islet beta-cells [32] and that its prolonged activation promotes beta-cell apoptosis [33]–[36]. Because this process is a major contributor to beta-cell dysfunction in diabetes, we sought ways to inhibit iPLA_2_β as a means to preventing beta-cell apoptosis. Though FKGK11 in now commercially available, it has been reported to be 7-fold less potent than FKGK18 in inhibiting iPLA_2_ [29]. If the FKGK18 compound were an effective inhibitor of beta-cell iPLA_2_β, it would allow us to utilize it to prevent beta-cell apoptosis *in vivo*. In the present study, we therefore set out to characterize the ability of FKGK18 to inhibit iPLA_2_β using INS-1 cells overexpressing iPLA_2_β (OE), rodent myocardial preparations, and human pancreatic islets.

## Results

### 2.1. FKGK18 inhibits iPLA_2_β similar to S-BEL

**Figure 2:**
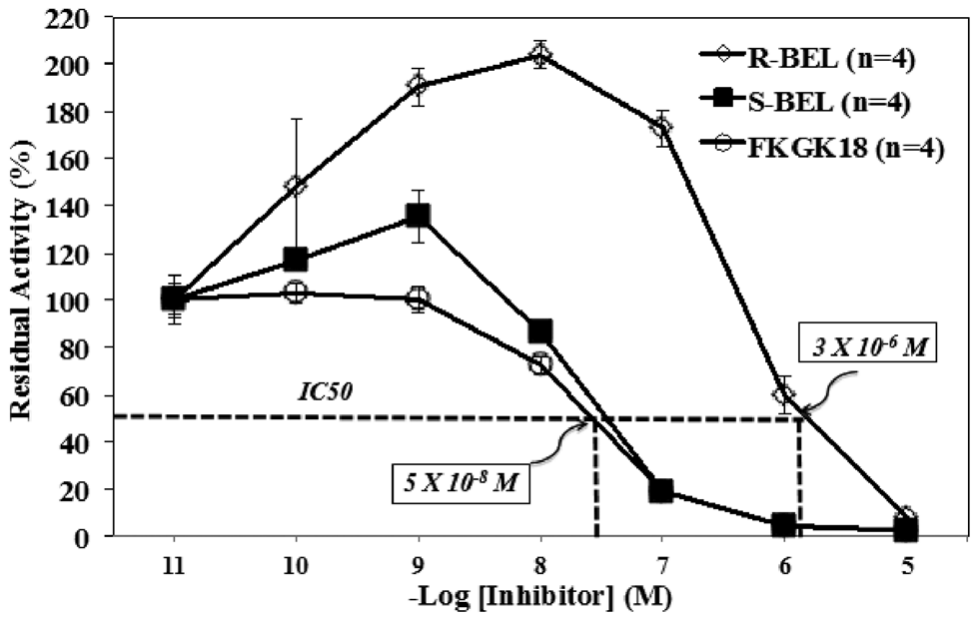
Comparison of Inhibition of Cytosol-Associated iPLA2β by BEL and FKGK18 in INS-1 OE Cells. Cytosol was prepared from INS-1 OE cells and the ability of FKGK18 to inhibit cytosol-associated iPLA_2_β activity was compared with that of *S*-BEL and *R*-BEL. The radioactivity enzymatic assay was performed using 30****µg protein aliquots and the data are presented as mean ± SEM of residual activity in the presence of an inhibitor, relative to activity measured in the presence of only the vehicle.

To test the ability of FKFK18 to inhibit iPLA_2_β activity, INS-1 insulinoma cells overexpressing iPLA_2_β (OE) were used. Cytosol from the OE cells was prepared and activity in 30****µg protein aliquots was assayed in the presence of varying concentrations of FKGK18. As seen in **Fig. 2**, FKGK18 inhibited Ca^2+^-independent PLA_2_ activity in a concentration-dependent manner, similar to *S*-BEL, which preferentially inhibits cytosol-associated iPLA_2_β [22]. The calculated IC50 (∼5×10^−8^ ****M) for FKGK18 was similar to that of *S*-BEL [6]. In contrast, *R*-BEL, which inhibits membrane-associated iPLA_2_γwas a weaker inhibitor of iPLA_2_ enzymatic activity, as reflected by an estimated IC50 of 3×10^−6^ ****M. These findings suggest that FKGK18 is equipotent to *S*-BEL as an *in vitro* inhibitor of cytosol-associated Ca^2+^-independent PLA_2_ activity, which is manifested by iPLA_2_β.

**Figure 3:**
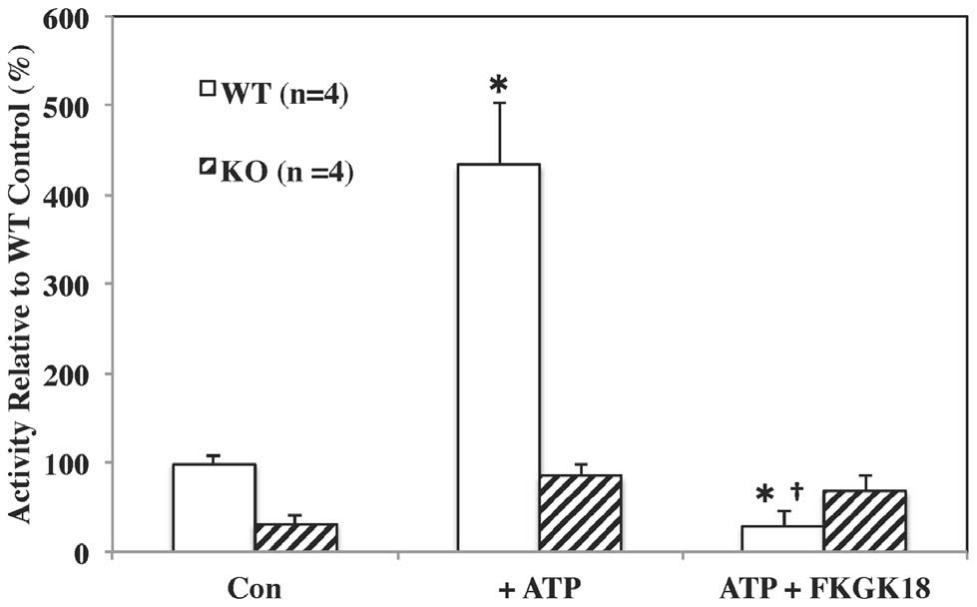
Cytosol-Associated iPLA_2_β Activity in Myocardium of WT and iPLA_2_β-KO Mice. Cytosol was prepared from hearts isolated from wild type (WT) and iPLA_2_β-deficient (KO) mice. The iPLA2β enzymatic radioactivity assay was preformed using 30****µg protein aliquots in the absence and presence of ATP (10****mM) and FKGK18 (10^−6^ ****M) either alone or in combination as indicated. The data for each group are presented as mean ± SEM of fold-change in activity in the presence of an inhibitor, relative to activity measured in the presence of only vehicle. (*Significantly different from WT Control group, p<0.05 and ^†^significantly different from WT+ATP group, p<0.05).

To confirm FKGK18 ability to inhibit iPLA_2_β, cytosol was prepared from myocardial tissues isolated from WT and iPLA_2_β-KO mice [37]. As illustrated in **Fig. 3**, cytosol-associated Ca^2+^-independent PLA_2_ activity in the WT group is stimulated by ATP, a characteristic of iPLA_2_β [32], nearly 5-fold and such stimulation was inhibited by FKGK18. In contrast, Ca^2+^-independent PLA_2_ activity ± FKGK18 in the iPLA_2_β-KO group was barely above background. These findings suggest that the myocardial cytosol-associated activity is manifested by iPLA_2_β and that it is inhibitable by FKGK18.

### 2.2. FKGK18 inhibits membrane-associated iPLA_2_ activity similar to R-BEL

**Figure 4:**
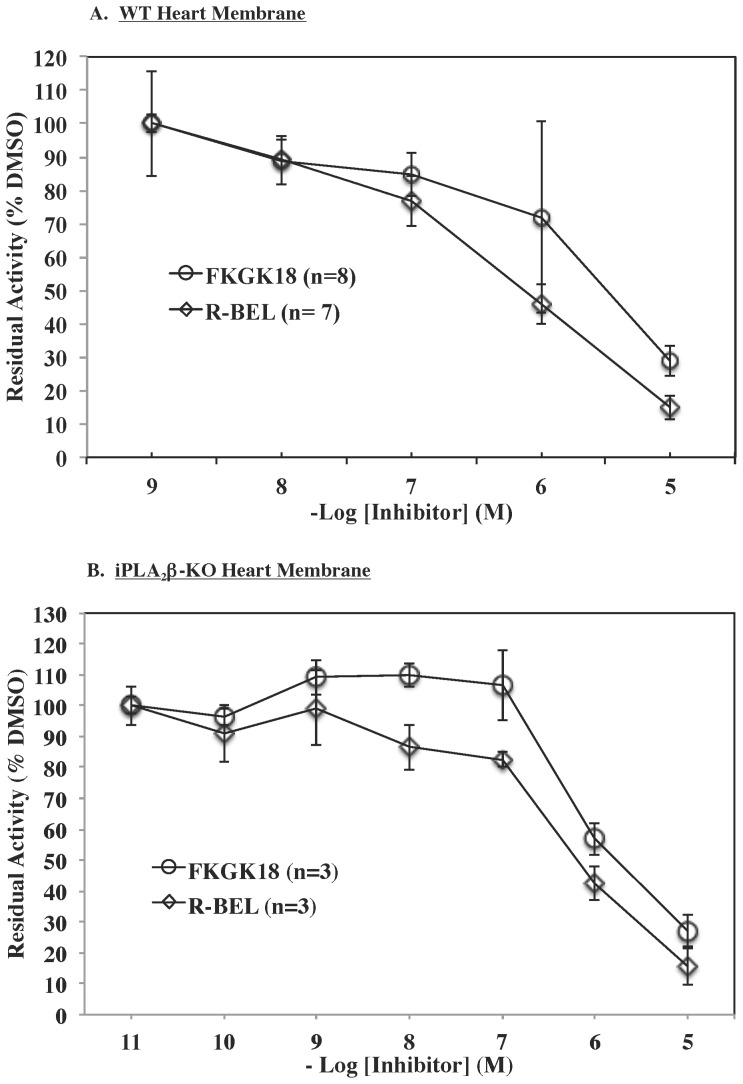
Inhibition of Membrane-Associated iPLA_2_ Activity in Hearts from WT and iPLA_2_β-KO Mice by *R*-BEL and FKGK18. Membrane fractions were prepared from hearts isolated from WT and iPLA_2_β-deficient (KO) mice and iPLA_2_β activity was assayed in 30****µg protein aliquots. The data are presented as mean ± SEM of residual activity in the presence of an inhibitor relative to activity measured in the presence of only vehicle. A. WT membrane-associated activity. Residual activity was assayed in the absence and presence of FKGK18, *S*-BEL, or *R*-BEL. B. KO membrane-associated activity. Residual activity was assayed in the absence and presence of FKGK18 or *R*-BEL.

Because the putative mechanism of FKGK18 inhibition is through interaction with the lipase consensus sequence of iPLA_2_, we examined whether FKGK18 also exhibited a similar inhibitory profile against iPLA_2_γ. We chose the myocardial membrane preparation because it is predominantly enriched in iPLA_2_γ activity [38]. Hearts from WT mice were isolated, membrane fraction prepared, and the inhibitory effects of *R*-BEL and FKGK18 on iPLA_2_γ activity were compared. As seen in **Fig. 4A**, activity is similarly inhibited in a concentration-dependent manner by *R*-BEL and FKGK18 with an IC50 of ∼1–3****µM. To confirm that the inhibition is of activity manifested by iPLA_2_γ, membrane fractions were prepared from hearts of iPLA_2_β-KO mice. As shown in **Fig. 4B**, FKGK18 inhibited membrane-associated iPLA_2_ activity similar to *R*-BEL, with an IC50∼1****µM. These findings suggest that iPLA_2_γ is also inhibitable by FKGK18.

### 2.3. Comparison of FKGK18 inhibition of cytosol- and membrane-associated iPLA_2_

**Figure 5:**
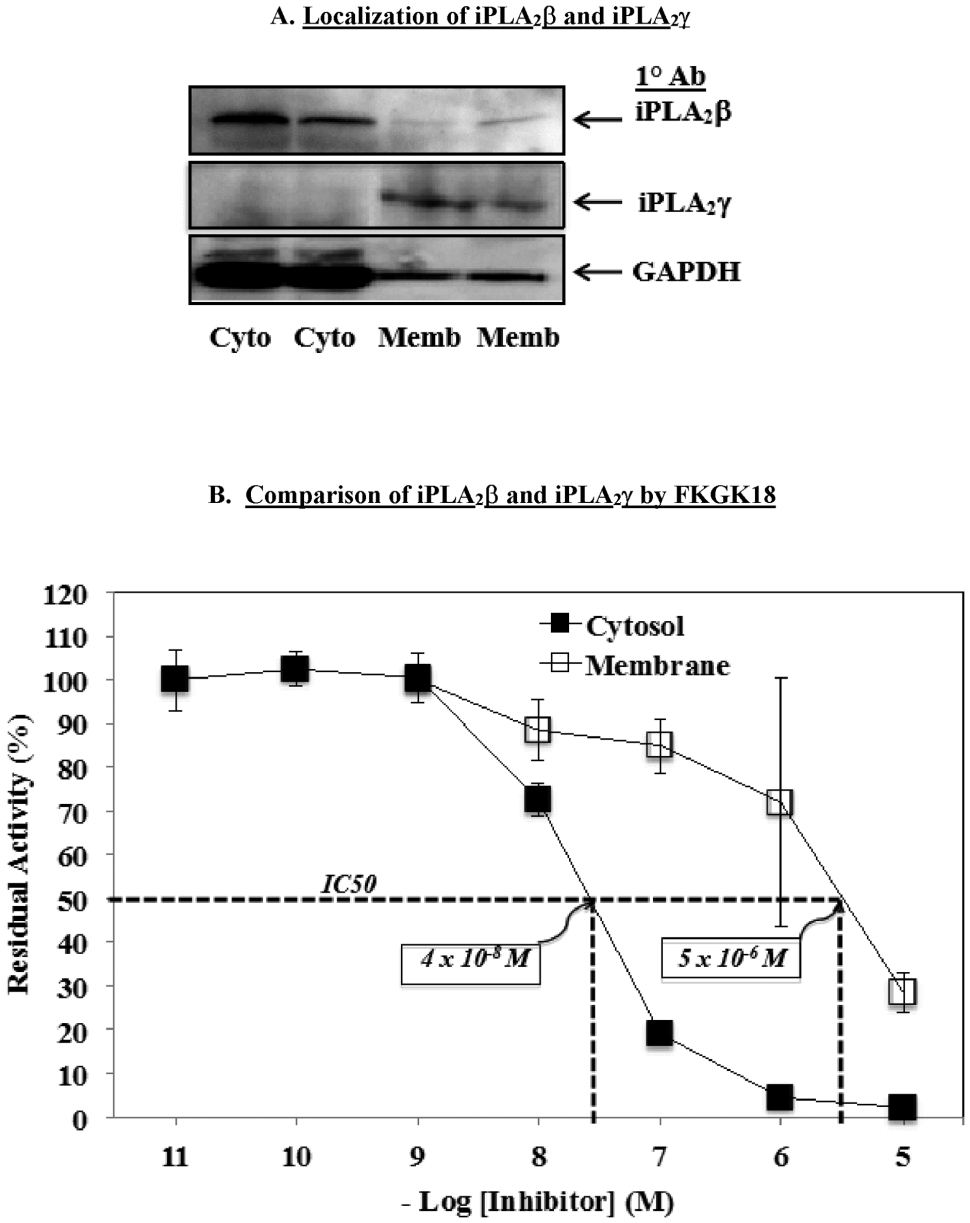
Inhibition of Myocardial Cytosol- and Membrane-Associated iPLA_2_ Activity by FKGK18. A. Organelle localization of iPLA_2_β and iPLA_2_γ. Cytosol and membrane fractions were prepared and processed for immunoblotting analyses using primary antibody against iPLA_2_β (Top panel), iPLA_2_γ (Middle Panel), and loading control GAPDH (Bottom Panel). B. Summary plots of residual activity in cytosol (iPLA_2_β) and membrane (iPLA_2_γ) the presence of FKGK18. Cytosolic and membrane fractions were prepared from WT hearts and iPLA_2_β activity was assayed in 30 µg protein aliquots. The data are presented as mean ± SEM of residual activity in the presence of the inhibitors expressed, relative to the activity measured in the presence of vehicle alone. The estimated IC50 of each is shown.

Though FKGK18 exhibited an ability to inhibit both iPLA_2_β and iPLA_2_γ, there was a distinct separation in the potency of the drug to inhibit the two activities. To verify localization that the cytosol and membrane preparations contained the expected isoform of iPLA_2_, cytosol and membrane preparations were processed for immunoblotting analyses using antibodies directed against iPLA_2_β or iPLA_2_γ. As shown in **Fig. 5A**, iPLA_2_β was predominantly localized in the cytosol (**Top Panel**) and iPLA_2_γ in the membrane (**Middle Panel**). Thus, the activities measured in cytosol and membrane fractions are expected to be manifested by iPLA_2_β and iPLA_2_γ, respectively. As illustrated in **Fig. 5B**, the FKGK18 inhibitory profile of cytosol-associated iPLA_2_β activity was shifted nearly two log-units to the left of membrane-associated iPLA_2_γactivity. The IC50 of FKGK18 for inhibition of cytosol-associated activity was nearly 100-fold lower than for membrane-associated activity, suggesting that FKGK18 is a more potent inhibitor of iPLA_2_β than iPLA_2_γ.

### 2.4. FKGK18 does not inhibit chymotrypsin like S-BEL

**Figure 6:**
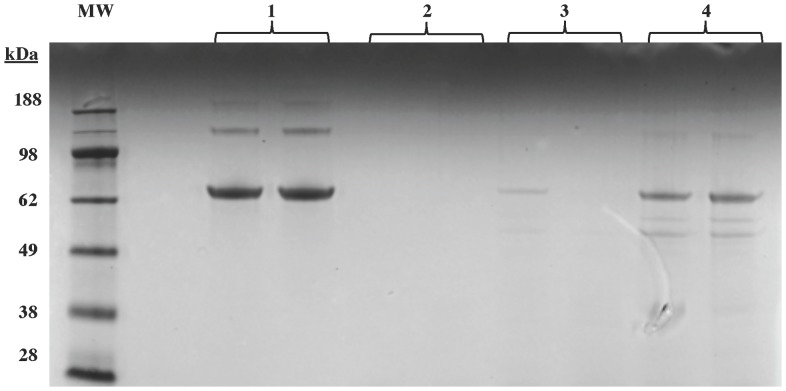
Comparison of *S*-BEL and FKGK18 Effects on Alpha-Chymotrypsin Activity. BSA (2****µg) was digested with trypsin (T) + α-chymotrypsin (C) (final concentration of each <100****nM) in the absence and presence of *S*-BEL and FKGK18 (20****µM) for 15****min at 37°C. The peptide digests were dried down to 20****µl and loaded onto 4–12% Bis-Tris gel and peptide fragments were separated for 35****min at 200V constant and visualized by overnight colloidal blue staining. Duplicate Lanes 1, *S*-BEL + FKGK18; 2, T + C; 3, T + C + FKGK18; and 4, T + C + *S*-BEL.

It has been reported that BEL inhibits other proteases [10], [11], [22] and that *R*-BEL is more potent than *S*-BEL [22]. To determine if FKGK18 non-specifically inhibits proteases, the ability of FKGK18 to inhibit α-chymotrypsin was compared with that of *S*-BEL. This was done by monitoring α-chymotrypsin-catalyzed digestion of BSA in the presence of saturating concentrations of *S*-BEL or FKGK18. The peptide digests were resolved by Bis-Tris gel electrophoresis and peptide fragments derived from α-chymotrypsin activity were visualized by colloidal blue staining. As shown in **Fig. 6** **(Lanes 1)**, in the presence of inhibitors alone a protein band corresponding to full-length BSA (70 kDa) is evident. However, in the presence of enzymes alone, BSA digestion in complete, as reflected by the absence of intact BSA (**Lanes 2**). Inclusion of FKGK18 with the enzymes also resulted in near completed digestion of BSA (**Lanes 3**). In contrast, inclusion of *S*-BEL with the enzymes, inhibited BSA digestion, as evidenced by visualization of the full length BSA (**Lanes 4**). These findings suggest that, unlike *S*-BEL, FKGK18 is not a non-specific inhibitor of non-iPLA_2_ proteases.

### 2.5. FKGK18 inhibition of iPLA_2_β is reversible

**Figure 7:**
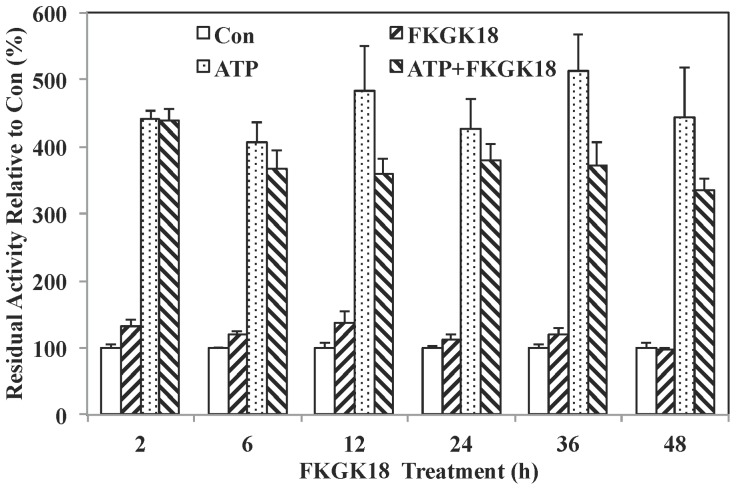
Temporal Effects of FKGK18 Exposure on iPLA_2_β Activity in INS-1 OE Cell Cytosol. INS-1 OE cells were treated with FKGK18 (10^−5^ M) for 2 to 48 h. Cytosol was then prepared and iPLA_2_β activity was assayed in 30 µg protein aliquots in the absence and presence of ATP (10 mM). The data are presented as mean ± SEM of activity, relative to that measured in Control groups.

Because BEL inhibition of iPLA_2_β is irreversible [19], we examined the duration of iPLA_2_βinhibition following a single exposure to FKGK18. INS-1 OE cells were treated with FKGK18 (10^−5^ M) for up to 48 h and cytosol was prepared and assayed for residual iPLA_2_β activity. As shown in **Fig. 7**, activity measured following FKGK18 exposure from 2 to 48****h was similarly stimulated by ATP, indicating preservation of a viable iPLA_2_β activity. However, neither basal nor ATP-stimulated activity in cytosol prepared from cells treated with FKGK18 from 2 to 48****hours was inhibited. These findings raise the possibility that the interaction of FKGK18 with the enzyme is disturbed during preparation of cytosol, thus restoring native enzyme activity, suggesting that FKGK18 is not an irreversible inhibitor of iPLA_2_β.

### 2.6. FKGK18 inhibits glucose-stimulated insulin secretion (GSIS) and PGE2 generation

**Figure 8:**
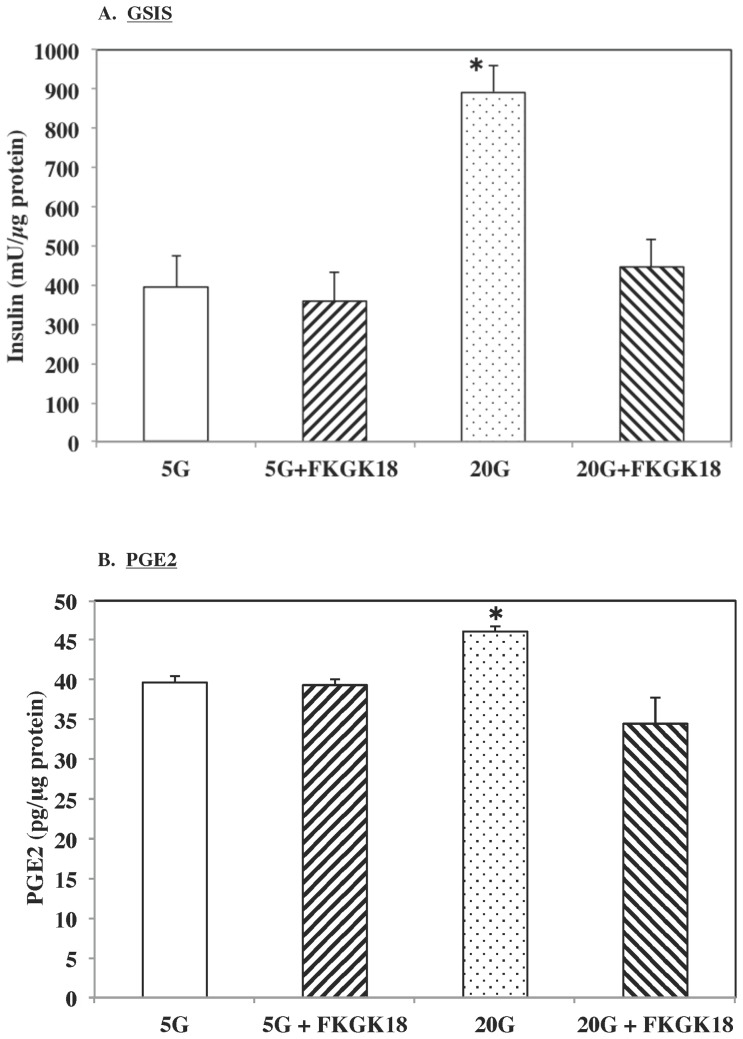
Effects of FKGK18 on Glucose-Stimulated Insulin Secretion (GSIS) and PGE2 Generation in Human Pancreatic Islets. Human pancreatic islets (30/condition) were incubated in KRB containing 5 mM glucose (5G) for 1 h at 37°C under 5%CO_2_/95% air atmosphere. The medium was then replaced with 5G + DMSO or 5G + FKGK18 (10^−6^ M) and the islets were incubated for 1 h. The islets were then exposed to KRB medium containing 5G+DMSO, 5G+FKGK18, 20G+DMSO or 20G+FKGK18. Medium was collected after 1 h and insulin and PGE2 contents in the medium were measured by ELISA. The islets were washed in PBS (3×) and islet protein concentration was determined. The data were normalized to total protein content. A. GSIS. (*20G group significantly different from other groups, p<0.01.) B. PGE2 generation. (*20G group significantly different from other groups, p = 0.001).

The reversible nature of inhibition raised the question of whether FKGK18 would be an effective inhibitor of biological processes in beta-cells that were previously demonstrated to be susceptible to inhibition by *S*-BEL. We previously reported that GSIS and hydrolysis of arachidonic acid (AA) from beta-cell membrane phospholipids, reflected by increases in medium content of PGE2, a metabolized product of AA, are inhibited by BEL. We therefore treated human islets with glucose in the absence or presence of FKGK18 and measured insulin secretion and PGE2 release into the media. As illustrated in **Fig. 8A**, insulin secretion was increased nearly 2.5-fold in the presence of 20****mM glucose (20G), relative to basal concentration of glucose (5****mM). In the presence of FKGK18, there was no change in basal insulin secretion. In contrast, stimulated insulin secretion was significantly decreased to basal levels. In parallel with GSIS, PGE2 content in the medium was significantly increased in the presence of 20G (**Fig. 8B**). This is consistent with glucose-stimulated PLA_2_β-catalyzed hydrolysis of arachidonic acid from beta-cell membranes and its metabolism to PGE2, as reported earlier [39]–[42]. As with GSIS, presence of FKGK18 did not inhibit PGE2 generation under basal conditions but significantly reduced stimulated PGE2 release into the media. These findings suggest that FKGK18 inhibits GSIS from pancreatic islets and AA hydrolysis from beta-cell membranes and importantly that FKGK18 can penetrate intact islets and the beta-cells contained within the islets.

### 2.7. FKGK18 inhibits ER stress-induced increase in neutral sphingomyelinase 2 (NSMase2)

**Figure 9:**
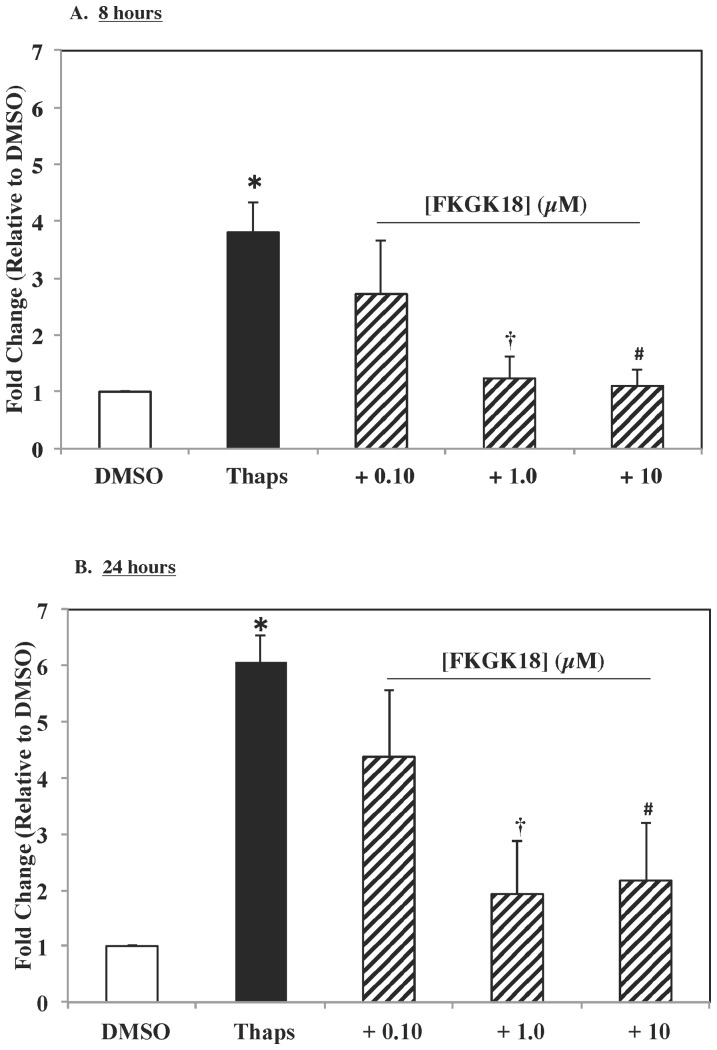
Effects of FKGK18 on ER Stress-Induced NSMase2 Expression in INS-1 OE Cells. INS-1 OE cells were treated with vehicle only or with thapsigargin (1 µM) for 8 or 24 h in the presence of FKGK18 (0.10, 1.0, or 10 µM). Total RNA was then prepared and cDNA generated and used to determine NSMase2 mRNA. The data are presented as fold-change in message, relative to vehicle-treated group only. A. 8 h. B. 24 h. (*Significantly different from vehicle group, p<0.001, **^†^**significantly different from Thaps group, p<0.003, and **^#^**significantly different from Thaps group, p = 0.03).

We previously also demonstrated that iPLA_2_β activation during ER stress induces NSMase in insulinoma cells and human and mouse pancreatic islet beta-cells by an iPLA_2_β-dependent mechanism and that this process participates in beta-cell apoptosis [33]–[35], [37], [43]. To examine if FKGK18 inhibits NSMase2 expression, we treated INS-1 OE cells with thapsigargin to induce ER stress in the absence and presence of FKGK18 and examined expression of NSMase2 message. Because our earlier work revealed near optimal increase in NSMase 2message and protein at 8–12 h and ER stress-induced beta-cell apoptosis at 24 h, we examined NSMase2 mRNA at 8 and 24 h. As previously observed, NSMase2 was induced by thapsigargin by 8 h (**Fig.** **9A**) and remained higher at 24****h (**Fig.** **9B**). Exposure to FKGK18 (pretreatment and treatment periods) promoted a concentration-dependent inhibition of NSMase2 expression at both time points. These finding suggest that FKGK18 inhibits ER stress-induced expression of NSMase2 in beta-cells.

### 2.8. FKGK18 inhibits ER stress-induced beta-cell apoptosis

**Figure 10:**
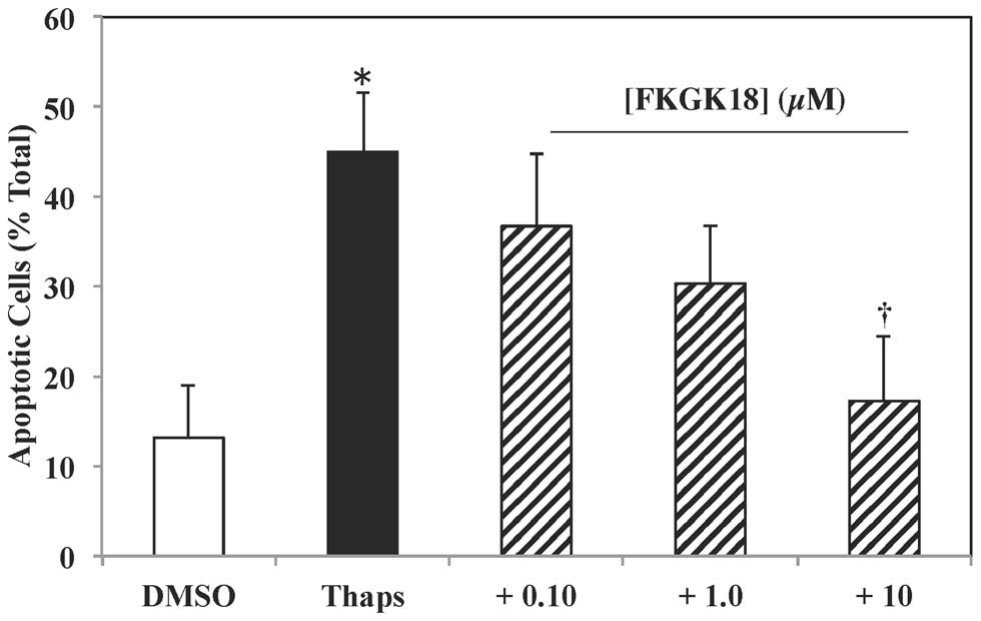
Effects of FKGK18 on ER Stress-Induced INS-1 OE Cell Apoptosis. INS-1 OE cells were treated with vehicle only or with thapsigargin (1****µM) for 24****h in the presence of FKGK18 (10^−7^–10^−5^ ****M). The incidence of apoptosis was then assessed by TUNEL staining using a flow cytometry protocol. The data in each group are plotted as fold-change in apoptosis, relative to vehicle treated cells. (*Significantly different from vehicle group, p<0.01 and †significantly different from Thaps group, p<0.05, n = 6).

Our collection of work reveals that iPLA_2_βplays a role in beta-cell apoptosis and chemical inhibition or knockdown of iPLA_2_βattenuates this process [33]–[35], [37], [43]. We therefore examined the ability of FKGK18 to prevent beta-cell apoptosis following induction of ER stress. INS-1 OE cells were pretreated with FKGK18 prior to and along with thapsigargin and flow cytometry analyses were used to assess the incidence of apoptosis, as reflected by TUNEL staining. Because in INS-1 OE cells apoptosis due to ER stress occurs between 20–24 h flow cytometry analysis was performed following exposure of the cells to thapsigargin for 24****h. As shown in **Fig. 10**, the incidence of apoptosis was significantly increased following induction of ER stress, relative to vehicle control. However, the presence of FKGK18 promoted a concentration-dependent decrease in ER stress-induced INS-1 OE cell apoptosis. These findings suggest that FKGK is an effective inhibitor of beta-cell apoptosis.

## Discussion

Presently, discerning of iPLA_2_-mediated effects by chemical means has been approached using BEL [19], [38]. However, feasibility of using BEL *in vivo* is limited by the irreversible nature of the inhibition by BEL along with its non-specific and potential cytotoxicity. Recently, compounds containing a fluoroketone (FK) group have been synthesized as potential inhibitors of the PLA_2_ enzymes. This led to the identification of FKGK18 (**Fig. 1**) as exhibiting the greatest potency to inhibit iPLA_2_ [29]. Furthermore, addition of a hydrophobic terminus connected by a medium-length carbon chain to mimic the fatty acid chain conferred selectivity of the FK compounds for iPLA_2_ versus the sPLA_2_ or cPLA_2_ isoforms [29]. However, these studies did not distinguish between inhibition of iPLA_2_β vs. iPLA_2_γ by FKGK18. Further, they did not test whether FKGK18 was an effective inhibitor of cellular iPLA_2_ activity or whether it impacted biological processes, previously described to involve iPLA_2_β activation.

Our group was the first to describe iPLA_2_β in the pancreatic islets and we found that it was predominantly expressed in the beta-cells of pancreatic islets [32], [43] and that it participated in glucose-stimulated insulin secretion (GSIS) [32]. Further studies indicated that long-term activation of iPLA_2_β contributes to beta-cell apoptosis [33], [36], [37], [43], raising the likelihood that iPLA_2_β activation participates in beta-cell death during the evolution of diabetes. Strengthening this possibility are the reports of increased iPLA_2_β expression in rodent models of diabetes and in human diabetes [44]–[46]. If we are to exploit the protective effects of inhibiting iPLA_2_β *in vivo*, it is necessary to utilize an inhibitor that is not irreversible or cytotoxic and we therefore set out to characterize the inhibitory profile of FKGK18 on iPLA_2_β in beta-cells.

Our findings comparing the cytosol-associated iPLA_2_ (iPLA_2_β) and the membrane-associated iPLA_2_ (iPLA_2_γ) activities in iPLA_2_βINS-1 OE cells and myocardial preparations from WT and iPLA_2_β-KO mice reveal that the potency of FKGK18 to inhibit iPLA_2_β is similar to the *S*-enantomer of BEL whereas the potency of FKGK18 to inhibit iPLA_2_γ is similar to the *R*-enantomer of BEL. However, FKGK18 inhibits iPLA_2_β with a greater potency than iPLA_2_γ; as reflected by the nearly 100-fold lower IC50 value for iPLA_2_β vs. iPLA_2_γ.

Attempts to determine the duration of FKGK18 inhibition of iPLA_2_βrevealed that even following exposure of INS-1 OE cells to the drug for 48 h, both basal and ATP-stimulated iPLA_2_β activities in cytosol prepared from these cells were similar to that measured in vehicle-treated cells. This is in contrast to the observation of concentration-dependent inhibition of iPLA_2_β activity when FKGK18 is added directly to cytosol preparations. Thus unlike with BEL, whose inhibition of iPLA_2_β activity in islets and INS-1 cells is close to 10–20% of control activity even after 24****h of exposure [47], the findings here suggest that FKGK18 inhibition of iPLA_2_β is reversible. This most likely is a consequence of disassociation of FKGK18 from iPLA_2_β during cell lysis in the cellular fractionation process. That this is not due to an inability of the FKGK18 to enter the cell is supported by the accompanying functional analyses in islets and cells, as discussed below.

We also examined for evidence of FKGK18 inhibition of α-chymotrypsin, which has been identified as a suitable target for aromatic haloenol lactones resulting in its mechanism-based inhibition [48]–[51]. In prior studies, *R*-BEL was determined to be a more potent inhibitor of chymotrypsin than its chiral counterpart *S*-BEL [22], [51]. Here, we observed that *S*-BEL nearly completely inhibited α-chymotrypsin-catalyzed digestion of BSA. In contrast, α-chymotrypsin-catalyzed digestion of BSA was nearly complete in the presence of FKGK18. These findings suggest that FKGK18 is not an effective inhibitor of α-chymotrypsin and imply that in contrast to BEL, it is not a non-specific inhibitor of proteases.

The mechanism of FKGK18 inhibition of iPLA_2_ appears to be distinct from that of BEL. With respect to BEL, Cys651 alkylation is the covalent modification of iPLA_2_β that is responsible for loss of activity, and the alkylating species is a diffusible hydrolysis product of BEL rather than a tethered acyl-enzyme intermediate [28]. In contrast, computer modeling and deuterium exchange mass spectrometry reveal that FK compounds bind at the active lipase consensus site, mimicking binding of natural substrates [52]. The hydrophobic environment of the active site in iPLA_2_ favoring high affinity of the inhibitor presumably confers the greater specificity of FK compounds for iPLA_2_ versus non-iPLA_2_ enzymes.

In view of these findings, we next examined whether FKGK18 can inhibit biological processes in intact insulinoma cells and human pancreatic islets. Because FKGK18 appears to be a reversible inhibitor, we assessed its impact on biological outcomes that would reflect dynamic iPLA_2_β inhibition. These included: (a) GSIS, (b) hydrolysis of arachidonic acid (AA), as reflected by PGE2 generation, (c) ER stress-induced neutral sphingomyelinase 2 (NSMase2) expression, and (d) ER stress-induced beta-cell apoptosis. As discussed below, during our descriptions of iPLA_2_β role in beta-cell function and survival, we observed that these outcomes are all inhibited by BEL.

We demonstrated that GSIS from pancreatic islets parallels iPLA_2_β-catalyzed hydrolysis of AA from the *sn*-2 position of beta-cell membranes, which are enriched in AA-containing phospholipids [39], [40], and that both are inhibited by BEL. Glucose induces accumulation of unesterified arachidonate in islets but little of this is released into the medium [53]. However, the oxygenated arachidonate metabolite PGE2 is, and is therefore used to reflect AA accumulation in the islet [54]. Here, treatment of human islets for one hour with stimulating concentrations of glucose (20****mM) promoted significant increases in both insulin secretion and PGE2 release, relative to basal (5****mM) glucose condition. Initial studies followed the protocol previously used with BEL [41], where FKGK18 was present for only 30****min prior to the replacement of media containing higher glucose concentrations (20****mM). With this protocol, we found that both GSIS and PGE2 release were not affected (*data not shown*), further supporting the reversible nature of FKGK18 inhibition. However, when FKGK18 was present during the entire stimulatory period, both GSIS and PGE2 release were reduced significantly. It therefore seems plausible to deduce that the inhibitory effects of FKGK18 on iPLA_2_β, and thus effects on GSIS and PGE2 release, are conditional upon the constant presence and exposure to FKGK18.

We also reported that prolonged ER stress promotes beta-cell apoptosis via activation of iPLA_2_β, which induces NSMase2 and accumulation of ceramides as a consequence of increased hydrolysis of sphingomyelins [34]. To determine if FKGK18 attenuates these ER stress-related outcomes, INS-1 OE cells were treated with thapsigargin to induce ER stress in the absence or presence of FKGK18. In its absence, NSMase2 message is increased significantly by 8****h and remained higher at 24****h. Presence of FKGK18 promoted a concentration-dependent decrease in NSMase2 message at both time points. Because NSMase2 increase is an early event and apoptosis is a later event, the incidence of apoptosis was assessed at 24****h. Thapsigargin, in the absence of FKGK18 promoted INS-1 OE beta-cell apoptosis, as reflected by increased TUNEL staining. However, in the presence of FKGK18 a concentration-dependent decrease in the incidence of apoptosis was evident. These findings are analogous to those observed in insulinoma cells and islet beta-cells, where both ER stress-induced NSMase2 expression and apoptosis were inhibited by BEL treatment [34], [43].

In summary, our studies reveal that FKGK18 is a more potent inhibitor of iPLA_2_βthan iPLA_2_γ and because, unlike BEL, it inactivates iPLA_2_β reversibly and appears to not be a non-specific inhibitor of proteases, FKGK18 may be the inhibitor of choice for *in vitro*, *ex vivo* and more importantly, *in vivo* studies. It is recognized that FKGK18 may have effects on other classes of non-PLA_2_ enzymes and continued studies with this and future FK derivatives will reveal other, if any, non-specific effects of these compounds. The ability to identify an inhibitor of iPLA_2_β that can be used *in vivo* is timely and warranted because of the ever going recognition of roles for iPLA_2_β-derived products in biological processes in the context of diabetes, inflammation, and neurodegenerative and myocardial disorders. It is plausible that FKGK18 or newer generations of FK compounds could be used to prevent or delay abnormalities associated with these disease states.

## Materials, Methods, and Experimental Procedures

### 4.1. Ethics Statement

Mice (WT and iPLA_2_β-KO breeding pairs), generously provided by Dr. John Turk (Washington University School of Medicine) were bred, maintained, and prepared for experiments according to the protocols approved by University of Alabama at Birmingham IACUC (APN# 120809160).

### 4.2. Reagents

Human islets were acquired from Islet Cell Resource Centers for Islet Distribution Program. FKGK18 (1,1,1-trifluoro-6-(naphthalen-2-yl)hexan-2-one) was synthesized, as previously described [29]. Other materials and (source) were as follows: *L*-a-1-palmitoyl-2-arachidonyl- [arachidonyl-1-^14^C] (PAPC, 58.2 mCi/mmol) (Amersham, Arlington Heights, IL); PNPLA8 antibody (Antibodies Online, Atlanta, GA); Apoptosis Detection Kit [APO-Direct^TM^] (BD Biosciences, San Jose, CA); *S*- and *R*-BEL, prostaglandin E2 EIA Kit – Monoclonal # 514010 (Cayman Chemicals, Ann Arbor, MI); MIAMI Medium 1A (Cellgro, Mediatech, Inc); Bis-tris, colloidal blue staining kit, RPMI medium 1640, Fast SYBR^®^ Green PCR Master Mix, SDS sample buffer, and Superscript III First-strand Synthesis System (Life Technologies Corporation, Grand Island, NY); Human Insulin ELISA (Mercodia, Pittsburg, PA), chymotrypsin and trypsin (Promega, Madison, WI); RNeasy kit (Qiagen Inc, Valencia, CA); Primary antibodies [sc-14463, sc-25778] and secondary antibodies [sc-2030, sc-2350] (Santa Cruz, Dallas, TX); and BSA and Pierce ECL Western Blotting Substrate (Thermo Scientific, Prod.).

### 4.3. INS-1 cell and islet culturing and treatment

INS-1 cells overexpressing iPLA_2_β (INS-1 OE) were generated and cultured as previous described [36]. Briefly, cells were cultured in RPMI 1640 medium, containing 11****mM glucose, 10% fetal calf serum, 10****mM HEPES buffer, 2****mM glutamine, 1****mM sodium pyruvate, 50****mM mercaptoethanol (BME), and 0.1% (w/v) each of penicillin and streptomycin in cell culture conditions (37°C, 5%CO_2_/95% air), as described [55]. Medium was changed every 2 days and cells were split once a week. Human islets were assessed immediately upon receipt under a microscope, non-islet matter was removed, and the islets were cultured (37°C, 5%CO_2_/95% air) for two days prior to use. Islets were also isolated from WT and iPLA_2_β-KO mice, as described [37] and cultured (37°C, 5%CO_2_/95% air) overnight prior to use. All inhibitors tested were prepared in DMSO vehicle and all experimental protocols included DMSO-only treated replicates. Of note, FKGK18 concentrations ≥5×10^−5^ ****M caused cells to detach from the bottom of wells and die and for this reason, the concentrations of drug used were below these.

### 4.4. iPLA_2_ enzyme activity assay

INS-1 OE cell or islet cytosol and membrane fractions were prepared and protein concentration determined using Coomassie reagent. Enzymatic Ca^2+^-independent PLA_2_ activity in aliquots of cytosol or membrane fractions (30****µg of protein) was assayed by ethanolic injection (5****µl) of the substrate 16:0/[^14^C]20:4 GPC (PAPC, 5****μM) in assay buffer (40****mM Tris, pH 7.5, and 10****mM EGTA, total volume 200****µl). Assay mixtures were incubated (3****min, 37°C, with shaking), and the assay reaction was terminated with butanol (100****µl) addition and vigorous vortexing. The reaction mixture was centrifuged (2,000×g, 5****min), and products in the upper butanol layer were analyzed by silica gel G thin-layer chromatography (TLC) in petroleum ether-ethyl ether-acetic acid (80/20/1). The TLC plate region containing free fatty acid was identified with iodine vapor, scraped, and the released ^14^C fatty acid was quantitated by liquid scintillation spectrometry. Specific iPLA_2_ activity was calculated from the dpm of released fatty acid and protein content, as described [32]. The assay was performed in the absence or presence of *S*- BEL, *R*-BEL, or FKGK (10^−11^–10^−5^ ****M). In some studies, to verify that the measured activity reflected that of iPLA_2_β, the ability of ATP (10 mM) to stimulate activity was used as a positive control.

### 4.5. iPLA_2_ immunoblotting analyses

Protein from cytosolic and microsomal membrane fractions was analyzed by SDS-PAGE; an equal protein load of 30 μg. The samples were run on a 10% acrylamide gel and then transferred onto Immunobolin-P PVDF membranes. Target proteins were probed with primary antibodies (iPLA2β1:200; iPLA_2_γ 1:200; and GAPDH 1:500) and then with secondary antibodies (1:1000). Immunoreactive bands were visualized by enhanced chemiluminescence.

### 4.6. Glucose-stimulated insulin secretion and PGE2 generation

Human islets (30/condition) were incubated in 96-well round bottomed plates in MIAMI medium, supplemented with 10% fetal calf serum and 0.1% (w/v) each of penicillin and streptomycin, as described [43]. The islets were pre-treated for 1****h with Krebs-Ringer buffer (KRB) containing 5****mM *D-*glucose (5G) in the presence of vehicle only or FKGK18 (10^−6^ or 10^−5^ M). Following pre-treatment, the medium was aspirated and islets were further treated for 1****h with 5G or 20G in the presence of vehicle or FKGK18. The medium was then collected and the contents of insulin and PGE2 were determined by ELISA. Briefly, collected medium was titrated against internal standard controls of known concentrations and standard curves were then generated to calculate sample unknown concentrations for insulin (using a log standard curve) and for PGE_2_ (using a 4-parameter logistics curve generated by ReaderFit software). The values were normalized to protein per sample (i.e. 30 islets) and are presented as mU/μg islet protein for insulin and pg/μg islet protein for PGE2.

### 4.7. Alpha-Chymotrypsin Catalyzed Cleavage Activity Assay

Bovine serum albumin (BSA, 2****µg) was reconstituted in 3 ul of 6 M Urea/50****mM Tris-HCl (pH 8). Reduction and alkylation was performed by adding 0.15****µl of 200 mM DTT/50 mM Tris-HCl (pH 8) for 1****h, and 0.6****µl of 200****mM iodoacetamide/50****mM Tris-HCl (pH 8) for 45****min in dark, respectively, at room temperature. Alkylation was quenched by adding 0.6****µl of 200****mM DTT/50****mM Tris-HCl (pH 8), and incubating for 15****min at room temperature. Twenty microliters of 1 mM CaCl_2_/50****mM Tris-HCl (pH 7.5) was added to dilute the urea concentration down to 0.6****M. Protease inhibitors FKGK18 and/or *S*-BEL were added to appropriate tubes. Samples were incubated with 0.2****µg of trypsin and 0.2****µg of chymotrypsin at 37°C for 15****min, and immediately quenched by denaturing in SDS sample buffer, as per manufacturers instructions. Even volumes of each samples were loaded and separated on a 10% Bis-tris gel and peptide fragments were separated for 35****min at 200V constant and visualized by overnight colloidal blue staining.

### 4.8. Quantitative Real-time PCR

INS-1 OE cells were treated with thapsigargin (1****µM) for 8 or 24****h in the presence of vehicle or FKGK18 (10^−7^–10^−5^ M). Total RNA was prepared using the RNeasy kit as previous [34], [35] and double stranded cDNA generated using the Superscript III First-strand Synthesis System, as described [37], [43]. Real-time PCR was performed with Fast SYBR^®^ Green PCR Master Mix in a plate-based LightCycler^®^ 480 System (Roche Life Sciences). The primers were designed based on published sequences for rat NSMase2 and 18S, Gene Bank #AB047002 and #X01117, respectively. Primer sets (sense/antisense) were as follows: NSMase2, ccggatgcacactacttcagaa/ggattgggtgtctggagaaca and 18S, agtcctgccctttgtacaca/gatccgagggcctcactaaac.

### 4.9. Apoptosis Assessment by TUNEL analyses

INS-1 OE cells were treated with vehicle or thapsigargin for 24****h in the absence or presence of FKGK18 (10^−7^–10^−5^ M). The cells were then processed for apoptosis as described [56] using the APO-DIRECT^TM^ kit, according to manufacturer’s protocol. Briefly, cells were harvested, spun at 800g and washed in PBS preceding the addition of fixation buffer (1% (w/v) paraformaldehyde in PBS (pH 7.4). Following a 30****min incubation period on ice, the cells were washed in PBS, resuspended in 70% ethanol, and incubated for a further 30****min on ice. DNA labeling solution (Reaction Buffer, TdT Enzyme, FITC dUTP and distilled water) was then added and the cells were incubated for 1****h at 37°C. After rinsing with rinse buffer and resuspension in PI/RNase staining buffer (provided in kit), cells were incubated in the dark for 30****min at room temperature prior to flow cytometry analysis at excitation wavelength 623****nm and 520****nm for PI (staining total DNA fragmentation) and FITC-dUTP (staining apoptotic cells), respectively. Of note, FKGK18 concentrations ≥5×10^−5^ ****M caused cells to detach and die and for this reason, the concentrations of drug used in the various analyses were ≤5×10^−5^ ****M.

### 4.10. Statistical Analysis

Data were converted to mean ± standard error of the means and the Students’ t-test was applied to determine significant differences between two samples (p<0.05).
